# Determination of Phosphoethanolamine in Urine with HPLC-ICPMS/MS Using 1,2-Hexanediol as a Chromatographic Eluent

**DOI:** 10.1021/acs.analchem.3c01364

**Published:** 2023-05-22

**Authors:** Bassam Lajin, Walter Goessler

**Affiliations:** † Institute of Chemistry, ChromICP, University of Graz, Universitaetsplatz 1, 8010 Graz, Austria; ‡ Institute of Chemistry, Analytical Chemistry for the Health and Environment, University of Graz, Universitaetsplatz 1, 8010 Graz, Austria

## Abstract

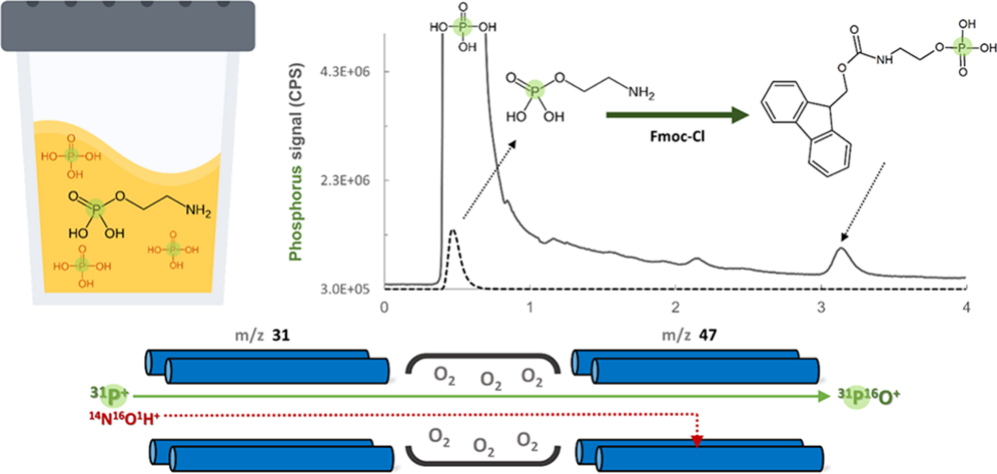

The importance of element-selective detection with inductively coupled plasma mass spectrometry (ICPMS) has been significantly increased in recent years following the introduction of tandem ICPMS (ICPMS/MS), which unlocked access to nonmetal speciation analysis. However, nonmetals are ubiquitous, and the feasibility of nonmetal speciation analysis in matrices with complex metabolomes is yet to be demonstrated. Herein, we report the first phosphorous speciation study by HPLC-ICPMS/MS in a human sample, namely, urine, involving the determination of the natural metabolite and biomarker phosphoethanolamine. A simple one-step derivatization procedure was employed to enable the separation of the target compound from the hydrophilic phosphorous metabolome in urine. The challenge of eluting the hydrophobic derivative under ICPMS-compatible chromatographic conditions was addressed by employing hexanediol, a novel chromatographic eluent recently described in our previous work but has not yet been exploited in a real-world application. The developed method features fast chromatographic separation (<5 min), no need for an isotopically labeled internal standard, and an instrumental LOD of 0.5 μg P L^–1^. The method was evaluated for recovery (90–110%), repeatability (RSD ±5%), and linearity (*r*^2^ = 0.9998). The method accuracy was thoroughly examined by comparing with an independently developed method based on HPLC-ESIMS/MS without derivatization, where agreement was found within ±5–20%. An application is presented to gain first insight into the variability in the human excretion of phosphoethanolamine, which is key for the interpretation of its levels as a biomarker, by repeated urine collection from a group of volunteers over 4 weeks.

## Introduction

Speciation analysis with the element-selective inductively coupled plasma mass spectrometry (ICPMS) as a chromatographic detector has been rapidly gaining recognition in various fields of research. However, phosphorous detection by ICPMS is limited by polyatomic interferences based on several species containing nitrogen, oxygen, and carbon, notably, ^14^N^16^O^1^H^+^, ^15^N_2_^1^H^+^, ^15^N^16^O^+^, ^14^N^17^O^+^, ^13^C^18^O^+^, and ^12^C^18^O^1^H^+^, which interfere with detection at *m*/*z* 31. This results in a high background signal due to the ubiquity of these interfering elements and therefore an increase in the detection limit. Furthermore, phosphorous speciation analysis using a single quadrupole ICPMS in highly complex matrices such as urine is particularly challenging due to the high abundance of organic compounds containing the above-mentioned interfering elements and the associated formation of polyatomic interferences, which could result in the appearance of chromatographic peaks not attributed to phosphorous-containing species and compromise the selectivity of detection. Indeed, the majority of phosphorous speciation studies performed with a single quadrupole ICPMS involved simple environmental matrices.^1−3^ Although polyatomic interferences at *m*/*z* 31 can be mitigated through the use of a collision/reaction cell,^3,4^ the most effective solution providing the highest selectivity is the employment of a triple quadrupole ICPMS (ICPMS/MS), where a reaction cell with oxygen as the reaction gas is used and the mass transition 31 → 47 is monitored using two quadrupole mass analyzers, achieving >10-fold improvement in the detection limit over single quadrupole ICPMS.^4−7^

Even though the advent of tandem mass spectrometry to ICPMS addressed the polyatomic interference issue resulting in lower detection limits and much higher selectivity, applicability of ICPMS/MS as a chromatographic detector for speciation analysis of a nonmetal such as phosphorous in biomedical research is still questionable due to the ubiquity of phosphorous in biological matrices and the high complexity of the phosphorous metabolome, which renders chromatographic separation a major challenge. Specifically, phosphorous is attached to a wide variety of metabolites in human matrices through enzymatic phosphorylation reactions, which greatly complicates targeting a minor phosphorous-containing metabolite with element-selective detection. A relatively small number of applications have been reported for chromatographic detection with ICPMS/MS involving phosphorous-containing compounds. These applications mostly involved the determination of organophosphorus herbicides in nonbiological matrices with simple phosphorous metabolomes^5,7,8^ or phosphorous-containing biomolecules in pure standard solution.^4^

Phosphoethanolamine is an essential building block for key phospholipids, notably phosphatidylethanolamine, which is implicated in a variety of medical conditions.^9^ In particular, phosphoethanolamine has been reported as a reliable biomarker with consistently elevated urinary levels in hypophosphatasia, an inherited metabolic bone disorder,^10^ with specificity and sensitivity of 100% and 88%, respectively.^11^ Application of phosphoethanolamine as a biomarker was also reported in other medical conditions than hypophosphatasia.^12^ The aim of the present work was to demonstrate that phosphorous speciation analysis in a highly complex matrix such as human urine can be made possible through a derivatization reaction with a hydrophobic reagent, which helps distinguish the target compound from the hydrophilic, and therefore urine-excretable, pool of phosphorous metabolites. A method for the determination of phosphoethanolamine with HPLC-ICPMS/MS is developed and applied to gain insight into the unexplored variability in the background urinary excretion of phosphoethanolamine in healthy volunteers.

## Experimental Section

### Urine Collection

The intra- and interindividual variability in the human urinary excretion of phosphoethanolamine was investigated by collecting morning first-pass urine samples from eight volunteers (mean age ± SD: 37 ± 13 years old; 5 males and 3 females) repeatedly over 4 weeks. The samples were collected on polypropylene containers (Corning, Corning Life Sciences GmbH, Germany), divided into portions (5 mL each), and stored at −80 °C until analysis. Urine collection was approved by the ethical committee at the University of Graz (GZ: 39/46/63).

To minimize the effects of variable fluid intake and enable correct assessment of the intra- and interindividual variability in the urinary excretion of phosphoethanolamine, concentrations were adjusted according to urine-specific gravity determined with a Leica TS 400 total solids refractometer (Leica Microsystems, Buffalo, NY).

### Sample Preparation and Derivatization

A one-step derivatization reaction was employed to distinguish phosphoethanolamine from the urine matrix. Derivatization was performed in a 1.5 mL Eppendorf tube by mixing 30 μL of untreated urine, 200 μL of the derivatization reagent Fluorenylmethyloxycarbonyl chloride (fmoc-cl) prepared at 25 g L^–1^ in acetonitrile, and 770 μL of sodium tetraborate buffer 0.1 M pH = 10.5 (adjusted with sodium hydroxide). The reaction mixture was vortex-mixed intermittently during 20 min of incubation at room temperature and then centrifuged. The supernatant was transferred to amber glass HPLC vials, and 30 μL was injected into the HPLC system.

### Quantification by HPLC-ICPMS/MS

Quantification by HPLC-ICPMS/MS was performed using an Agilent 1100 chromatographic system (Agilent Technologies, Waldbronn, Germany) consisting of a sample cooler (G1330B), a quaternary pump (G1311A), an autosampler (ALS G1367C), a degasser (G1379A), and a column compartment (G1316A). The chromatographic column (YMC Triart C18, 50 mm × 2.1 mm, 1.9 μm particle size) was connected using PEEK tubing (0.127 mm I.D and ca. 40 cm in length) to an Agilent 8900 ICPMS/MS system consisting of a quartz plasma torch with an inner diameter of 2.5 mm, an AriMist PEEK nebulizer, a glass Scott double pass spray chamber, and a Ni/Cu sampler and skimmer cones. Oxygen was employed as the reaction cell gas at 0.3 mL min^–1^ to produce the mass transition 31 → 47, which was used to detect phosphorous. Key ICPMS/MS instrumental settings were as follows: RF power 1550 W; RF matching: 1.7 V; sampling depth: 5.0 mm; nebulizer gas 0.65 L min^–1^; makeup gas 0.35 L min^–1^; and spray chamber temperature: 2 °C. All concentrations were reported based on phosphorous mass per volume (μg P L^–1^).

Isocratic chromatographic separation was performed using a mobile phase containing 9% v/v of 1,2-hexanediol (Sigma-Aldrich, Vienna, Austria) and 0.1% v/v acetic acid with pH adjusted to 9.5 with ammonia (ACS grade, Sigma-Aldrich, Vienna, Austria). The mobile phase flow rate was set at 0.25 mL min ^–1^, and the column temperature was held at 40 °C.

### Evaluation of Method Accuracy by Comparing with HPLC-ESIMS/MS

A total of 16 urine samples were analyzed using a method based on HPLC-ESIMS/MS developed in-house without derivatization. An Agilent 1260 Infinity II LC system (Agilent Technologies, Waldbronn, Germany) was employed for chromatographic separation, consisting of a quaternary 1260 Infinity II Flexible Pump (G7104C, max. pressure 800 bar), Multisampler (G7167A), and Multicolumn Thermostat (G7116A). A mobile phase containing 0.05% v/v acetic acid (pH adjusted to 9.5 with ammonia) was used at a flow rate of 0.5 mL min^–1^ with a Hamilton PRPX-100 (250 mm × 2.1 mm, 5 μm particle size) column held at a column temperature of 40 °C. A triple quadrupole Ultivo LC/TQ system (G6465B) was used for molecular tandem mass spectrometric detection, with the following instrumental parameters: mass transition 140 → 79; collision energy: 20 eV; capillary voltage: −3000 V; fragmentor voltage: 50 V; gas temperature: 350 °C; gas flow: 10 L min^–1^; sheath gas temperature: 400 °C; and sheath gas flow 12 L min^–1^. To account for matrix effects, quantification was performed using standard addition.

## Results and Discussion

**Figure 1:**
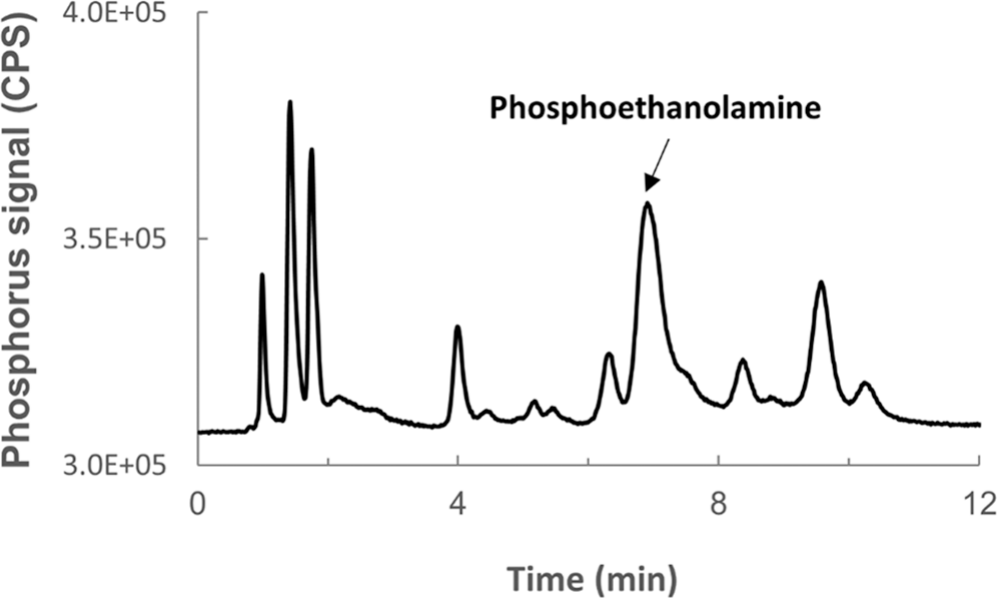
Chromatogram of an underivatized urine sample showing the complex phosphometabolome in urine with ICPMS/MS detection. Coelution was used to identify phosphoethanolamineat RT ca. 7 min. Chromatographic column: anion-exchange Hamilton PRP-X100 (250 mm × 2.1 mm, 5 μm particle size). Mobile phase: 7 mmol L^–1^ ammonium acetate pH = 9.0. Mobile phase flow rate: 0.6 mL min ^–1^. Column temperature: 50 °C. Injection volume: 1 μL.

Phosphoethanolamine (PEt) is unretained on reversed-phase columns, and attempts at separating the compound from the many phosphorous compounds in urine using ion-exchange chromatography were not successful due to the encountered complexity of the phosphorous metabolomic profile in urine under ICPMS/MS detection (Figure 1), which is a major challenge in phosphorous speciation analysis in biological samples. While many biometabolites can be phosphorylated, a relatively smaller number of these contain a primary amino group. Notable examples are the amino acids phosphoserine and phosphothreonine, but these are excreted in human urine at very low concentrations (0.1–10 μg P L^–1^).^13,14^ Therefore, to separate PEt from phosphorous-containing compounds in urine, a derivatization reagent targeting the primary amino group was employed.

**Figure 2:**
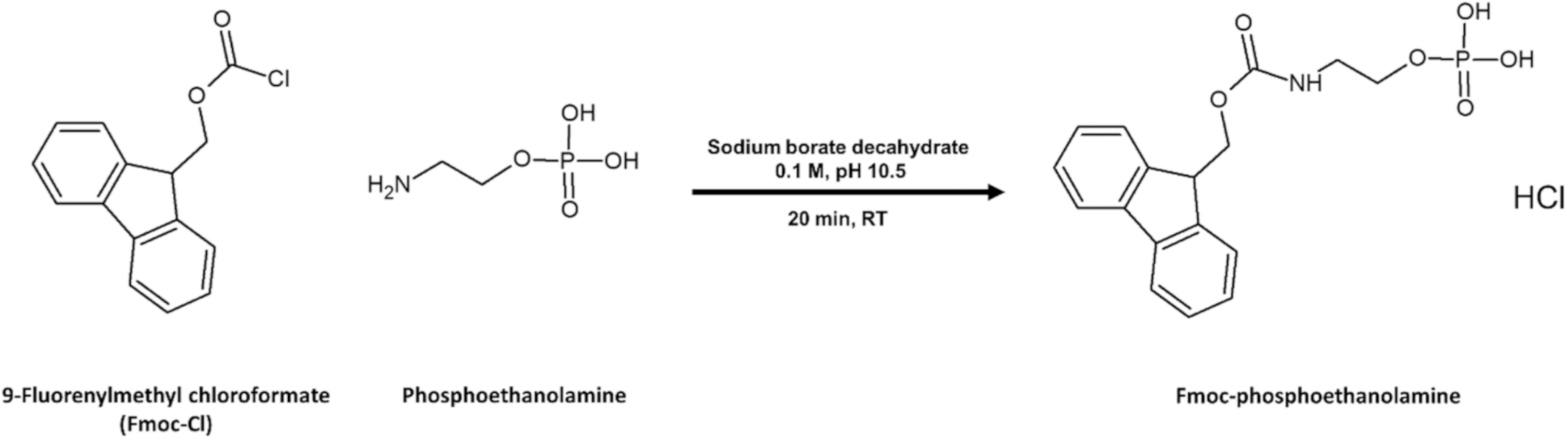
Reaction scheme for the derivatization of phosphoethanolamine. For reaction conditions, see the Experimental Section.

Fluorenylmethyloxycarbonyl chloride (fmoc-Cl) is commonly employed for the derivatization of primary amines and amino acids under basic pH conditions.^15^ A simplified procedure, including one-step derivatization, was sufficient to convert PEt to its fmoc derivative (Figure 2). The high hydrophobicity of the fmoc-PEt derivative enables separation from the hydrophilic, and therefore urine-excretable, phosphorous-containing metabolites. Moreover, the fmoc-PEt derivative is hydrophobic enough to be well separated from inorganic phosphate, which is present in human urine at concentrations higher than PEt by more than three orders of magnitude (>1 g L^–1^), eliminating the need for a phosphate removal step via precipitation.

However, the high hydrophobicity of the formed derivative results in strong retention and long retention time and would necessitate the incorporation of high percentages of organic eluents in the mobile phase. High organic content of the mobile phase results in plasma shutdown, and to address this incompatibility, the organic ICPMS mode would need to be engaged, which would have a negative impact on the limit of detection (e.g., through mobile phase flow rate splitting and post-column dilution). The negative impact of the organic ICPMS mode is clearly applicable to all elements, but the most common example in the literature is found in arsenic speciation analysis, where typical detection limits for the hydrophobic arsenolipids^16,17^ are >100-fold higher than those reported for hydrophilic arsenic species such as dimethylarsinic acid.^18^

**Figure 3:**
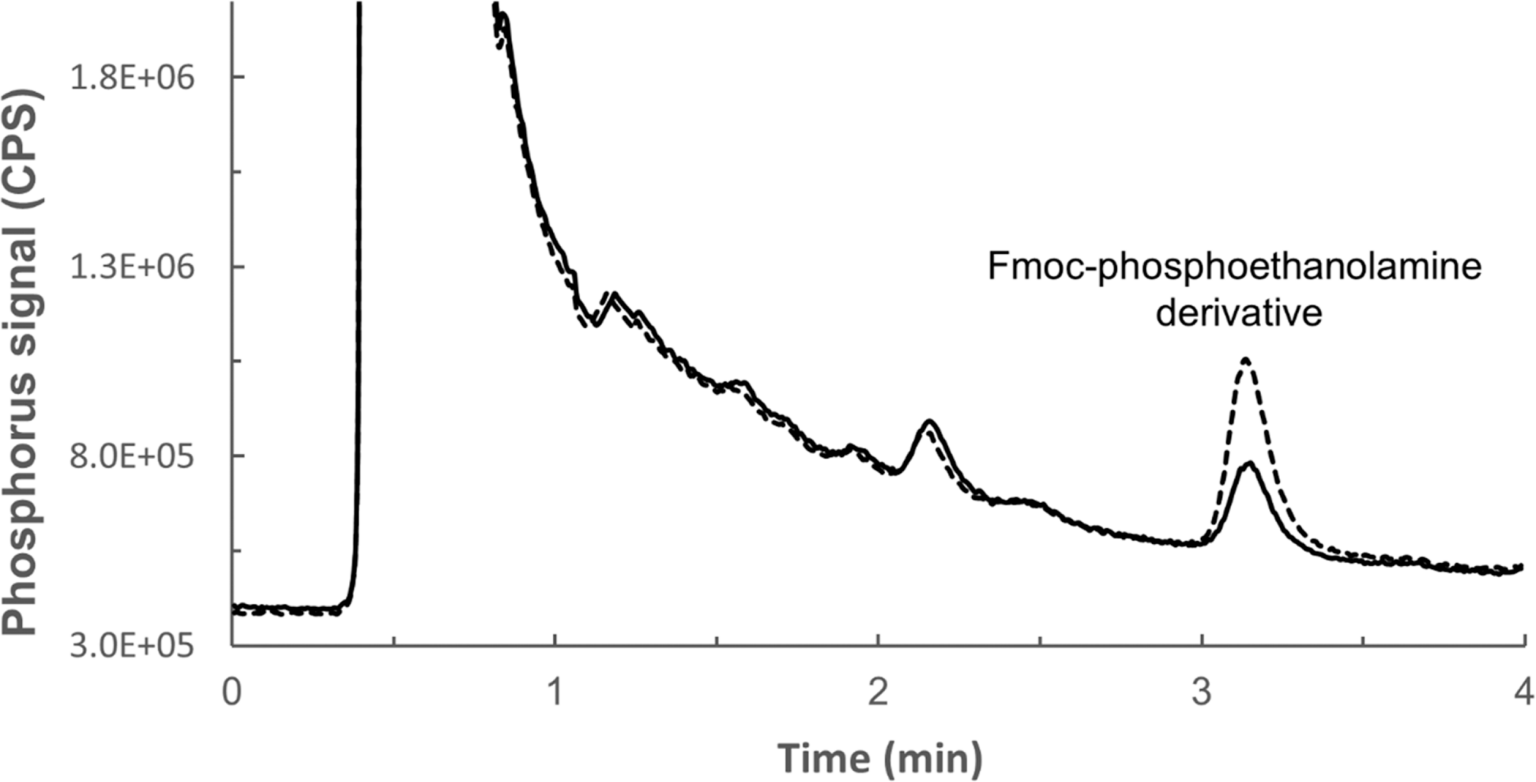
Chromatographic separation of the Fmoc-PE derivative from the urine matrix and detection with ICPMS/MS at mass transition 31 → 47. The solid line indicates unspiked urine, and the dashed line indicates urine spiked with 1.0 mg P L^–1^ of phosphoethanolamine prior to derivatization. The large front peak is attributable to inorganic phosphate, which elutes at the void time from the employed reversed-phase column. Phosphate dominates the phosphorous profile in human urine with concentrations 1.0–10 g L^–1^.

In order to avoid the organic ICPMS mode, we employed 1,2-hexanediol, a novel chromatographic eluent with remarkable properties, including exceptional elution strength and plasma tolerability, recently described in our previous work^19^ but has not yet been put into practice. As little as 9% v/v was sufficient to elute the hydrophobic derivative under standard ICPMS conditions and instrumental setup without employing any of the components of the organic ICPMS mode while achieving fast separation within <4 min (*k* = 7) (Figure 3). The instrumental limit of detection was 0.5 μg P L^–1^, which is similar to previously reported limits of detection for hydrophilic phosphorous-containing analytes with ICPMS/MS detection.^6−8^

The method limit of detection was 17 μg P L ^–1^, which is 34-fold higher than the instrumental limit of detection due to the dilution factor for urine (30 μL → 1000 μL total reaction volume). Since urine is an interindividually variable matrix, a high dilution factor was chosen to ensure method robustness and complete derivatization. The urinary concentrations of phosphoethanolamine measured in the present study were well above the limit of detection in all 32 urine samples (see below).

While tandem mass spectrometry addressed the issue of polyatomic interferences and enabled detection of nonmetals at <1.0 μg L^–1^, nonmetal speciation analysis with HPLC-ICPMS/MS in biological samples is particularly challenging due to the ubiquity and high complexity of the metabolome of the nonmetals, especially phosphorous and sulfur. Therefore, as shown in the present work, derivatization can be a requirement for detecting metabolites tagged with a nonmetal in such highly complex matrices using ICPMS/MS detection. In this respect, 1,2-hexanediol, which is an eluent recently described and tested in our previous work,^19^ is particularly advantageous in that it enables applying derivatization with hydrophobic reagents in speciation analysis with ICPMS/MS detection without the need for employing the inconvenient ICPMS organic mode, which would compromise the limit of detection and therefore offset the advantages of ICPMS/MS in nonmetal speciation analysis. In other words, the present work shows that coupling the use of derivatization with elution using hexanediol and detection with ICPMS/MS is an effective general strategy for nonmetal speciation analysis in complex biological matrices.

**Figure 4:**
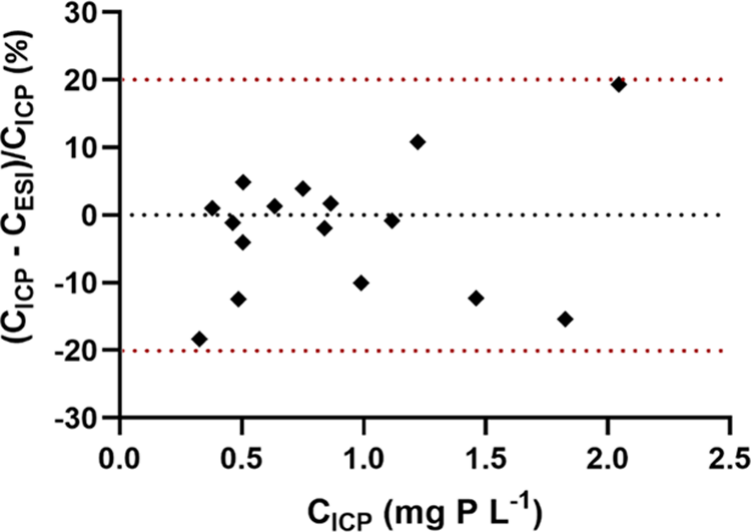
Evaluating the accuracy of the developed HPLC-ICPMS/MS method for the determination of phosphoethanolamine in urine with an independently developed method involving HPLC-ESIMS/MS applied without derivatization using standard addition. The graph shows a plot of the concentrations found with ICPMS/MS detection (*C*_ICP_) against the difference between the concentrations determined with the two methods normalized to the concentration determined with ICPMS detection (*C*_ICP_ – *C*_ESI_)/*C*_ICP_. The two methods were in agreement within ±20%.

Even though derivatization enabled complete separation of PEt from the urinary phosphorous metabolome (Figure 3), recovery experiments (Table 1) may not be sufficient to ensure selectivity due to the possibility of coeluting phosphorous-containing metabolites, given the high complexity of the urine matrix. Therefore, we evaluated the accuracy further using a second independently developed method employing HPLC-ESIMS/MS without derivatization. Comparing the concentrations found using the two methods shows agreement within less than ±20% for all 16 samples tested (Figure 4). A comprehensive table including concentrations measured with the two methods can be found in the Supporting Information (Supplementary Table S1).

**Table 1:** Recovery and Repeatability for the Determination of Phosphoethanolamine in Urine by HPLC-ICPMS/MS^a^

	L0	L1		L2	
sample	measured concentration (mg P L^–1^)	measured concentration (mg P L^–1^)	recovery (%)	measured concentration (mg P L^–1^)	recovery (%)
1	0.65 ± 0.02	1.57 ± 0.02	92 ± 4	5.1 ± 0.1	87 ± 1
2	0.52 ± 0.02	1.41 ± 0.01	90 ± 3	4.6 ± 0.1	81 ± 1
3	0.57 ± 0.01	1.62 ± 0.05	115 ± 6	5.4 ± 0.2	98 ± 4
4	0.87 ± 0.01	1.96 ± 0.02	109 ± 3	5.6 ± 0.1	95 ± 1
5	1.07 ± 0.07	1.88 ± 0.02	82 ± 5	4.6 ± 0.1	71 ± 1
6	0.50 ± 0.02	1.63 ± 0.01	113 ± 1	5.3 ± 0.2	96 ± 3

a The table displays the concentrations (mean ± SD) in a group of urine samples (*n* = 6) before (L0) and after spiking with phosphoethanolamine 1.0 mg P L^–1^ (L1) and 5.0 mg S L^–1^ (L2).

The most recent analytical methods for the determination of PEt are based on molecular tandem mass spectrometric detection (HPLC-ESIMS/MS), where instrumental limits of detection around 0.5–1 μmol L^–1^ (ca. 15–30 μg P L^–1^) have been reported.^13,20^ Therefore, the present HPLC-ICPMS/MS provides a clearly superior instrumental limit of detection at 0.5 μg P L^–1^ (0.02 μmol L^–1^) and has the advantage of much higher resistance to matrix effects commonly observed in heavy biological matrices with electrospray ionization-based mass spectrometry,^21^ which usually entails the employment of an isotopically labeled internal standard or the time-consuming method of standard addition with ESIMS methods. The present method, on the other hand, does not require the use of an isotopically labeled phosphoethanolamine and can be applied using simple external calibration.

**Figure 5:**
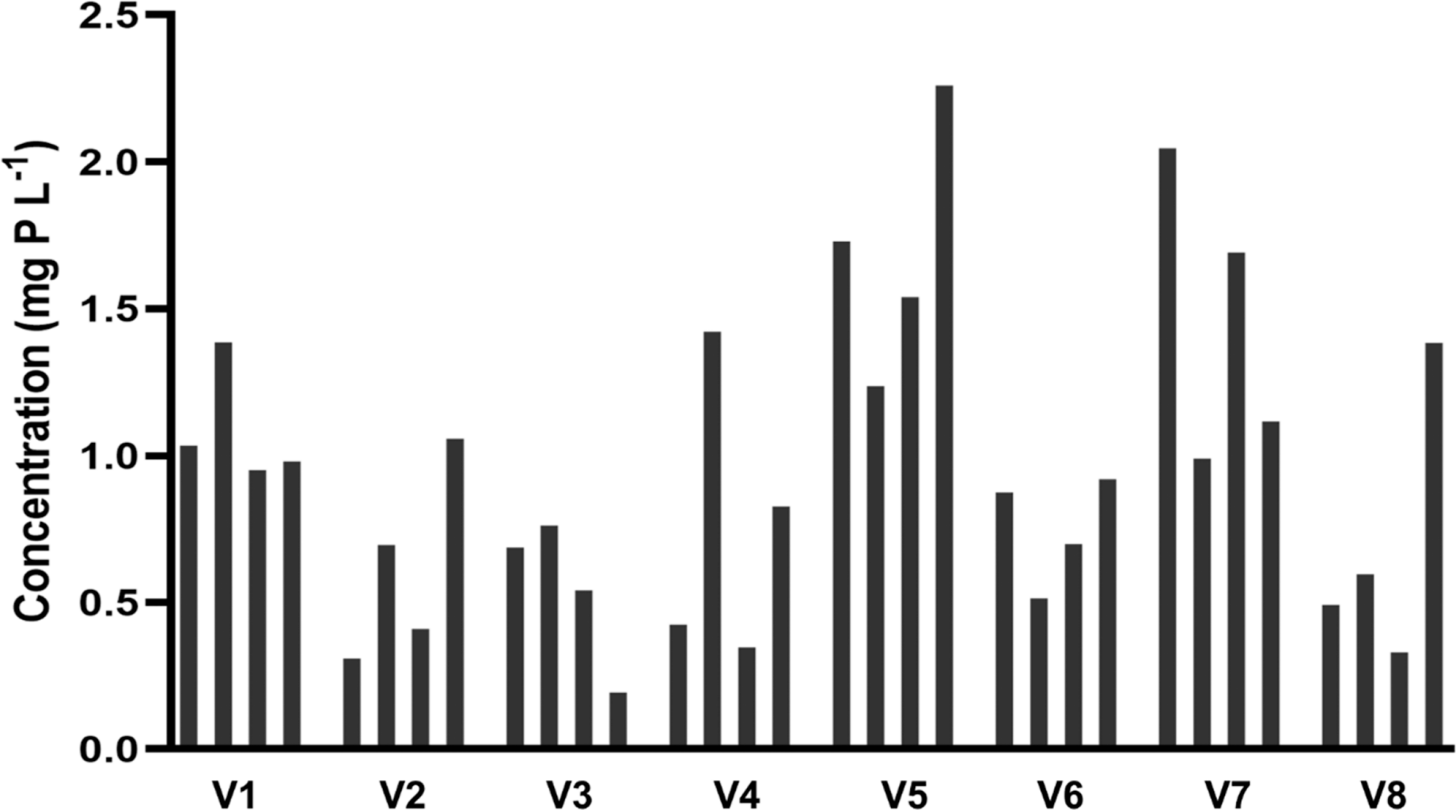
Applying the developed HPLC-ICPMS/MS method for the determination of phosphoethanolamine in human urine to investigate the inter- and intraindividual variability in the urinary excretion of the potential biomarker. Each group of columns represents concentrations of phosphoethanolamine in urine collected over 4 consecutive weeks from each of the eight recruited volunteers. Concentrations were adjusted according to specific gravity (see text) to minimize the influence of fluid on the observed patterns.

The utility of phosphoethanolamine as a biomarker largely depends on the inter- and intraindividual variability in its production. Since no systematic investigation of this topic has been found in the literature, the developed method was applied to morning first-pass urine samples collected repeatedly from eight healthy volunteers collected over a period of 4 consecutive weeks (for more details, see Experimental Section) in order to evaluate the biological variability in phosphoethanolamine excretion in urine. The mean ± SD for the measured urinary concentrations in the total population of samples (*n* = 32) was 0.93 ± 0.56 mg P L^–1^. After adjusting urinary concentrations using specific gravity to account for the influence of variable fluid intake, the inter- and intraindividually was <3-fold (Figure 5).

In conclusion, the present work highlights the applicability of element-selective ICPMS/MS detection for phosphorous speciation analysis in a human biological matrix with a complex phosphorous metabolome. The previously described advantages of 1,2-hexanediol as a strong organic eluent compatible with the inductively coupled plasma were shown in the present work to be specifically relevant to a derivatization approach, serving as a general strategy for nonmetal speciation analysis in biological samples.

## Supporting Information Available

Concentrations measured by HPLC-ICPMS/MS and HPLC-ESIMS/MS (Supplementary Table S1) (PDF)

## References

[ref1] Guo Z.-X.; Cai Q.; Yang Z. J. Chromatogr. A 2005, 1100, 160–167. 10.1016/j.chroma.2005.09.034. 16185703

[ref2] Guo Z.-X.; Cai Q.; Yang Z. Rapid Commun. Mass Spectrom. 2007, 21, 1606–1612. 10.1002/rcm.3003. 17443488

[ref3] Sadi B. B. M.; Vonderheide A. P.; Caruso J. A. J. Chromatogr. A 2004, 1050, 95–101. 10.1016/S0021-9673(04)01313-5. 15503930

[ref4] Fernández S. D.; Sugishama N.; Encinar J. R.; Sanz-Medel A. Anal. Chem. 2012, 84, 5851–5857. 10.1021/ac3009516. 22725632

[ref5] Lajin B.; Goessler W. Talanta 2019, 196, 357–361. 10.1016/j.talanta.2018.12.075. 30683376

[ref6] Pimenta E. M.; Silva F. F. d.; Barbosa É. S.; Cacique A. P.; Cassimiro D. L.; Pinho G. P.; de Silvério F. O. J. Braz. Chem. Soc. 2020, 31, 298–304.

[ref7] Tiago J. P. F.; Sicupira L. C.; Barros R. E.; Pinho G. P. de.; Silvério F. O. J. Environ. Sci. Health B 2020, 55, 558–565. 10.1080/03601234.2020.1733369. 32107966

[ref8] Fontanella M. C.; Lamastra L.; Beone G. M. Molecules 2022, 27, 8049 10.3390/molecules27228049. 36432148 PMC9696991

[ref9] Patel D.; Witt S. N. Oxid. Med. Cell. Longev. 2017, 2017, 1–18. 10.1155/2017/4829180. PMC5529665 28785375

[ref10] Whyte M. P. Nat. Rev. Endocrinol. 2016, 12, 233–246. 10.1038/nrendo.2016.14. 26893260

[ref11] Shajani-Yi Z.; Ayala-Lopez N.; Black M.; Dahir K. M. Bone 2022, 163, 116504 10.1016/j.bone.2022.116504. 35878747

[ref12] Rochmah M. A.; Wijaya Y. O. S.; Harahap N. I. F.; Tode C.; Takeuchi A.; Ohuchi K.; Shimazawa M.; Hara H.; Funato M.; Saito T. Kobe J. Med. Sci. 2020, 66, E1. 32814752 PMC7447103

[ref13] Kaspar H.; Dettmer K.; Chan Q.; Daniels S.; Nimkar S.; Daviglus M. L.; Stamler J.; Elliott P.; Oefner P. J. J. Chromatogr. B Analyt. Technol. Biomed. Life Sci. 2009, 877, 1838–1846. 10.1016/j.jchromb.2009.05.019. PMC2748257 19481989

[ref14] Kataoka H.; Nakai K.; Katagiri Y.; Makita M. Biomed. Chromatogr. 1993, 7, 184–188. 10.1002/bmc.1130070403. 7693088

[ref15] Jámbor A.; Molnár-Perl I. J. Chromatogr. A 2009, 1216, 3064–3077. 10.1016/j.chroma.2009.01.068. 19215925

[ref16] Khan M.; Francesconi K. A. J. Environ. Sci. 2016, 49, 97–103. 10.1016/j.jes.2016.04.004. 28007184

[ref17] Viczek S. A.; Jensen K. B.; Francesconi K. A. Angew. Chem. 2016, 128, 5345–5348. 10.1002/ange.201512031. PMC4949577 27478276

[ref18] Tanda S.; Ličbinský R.; Hegrová J.; Faimon J.; Goessler W. Sci. Total Environ. 2019, 651, 1839–1848. 10.1016/j.scitotenv.2018.10.102. 30317172

[ref19] Lajin B.; Feldmann J.; Goessler W. Anal. Chem. 2022, 94, 8802–8810. 10.1021/acs.analchem.2c01769. 35666989 PMC9218959

[ref20] Held P. K.; White L.; Pasquali M. J. Chromatogr. B 2011, 879, 2695–2703. 10.1016/j.jchromb.2011.07.030. 21852206

[ref21] Böttcher C.; Roepenack-Lahaye E. v.; Willscher E.; Scheel D.; Clemens S. Anal. Chem. 2007, 79, 1507–1513. 10.1021/ac061037q. 17297948

