# Supplementary material for: Determination of Phosphoethanolamine in Urine with HPLC-ICPMS/MS Using 1,2-Hexanediol as a Chromatographic Eluent
Source: Anal Chem. 2023 May 22;95(22):8706–10. doi: 10.1021/acs.analchem.3c01364 (PMC10248998; doi:10.1021/acs.analchem.3c01364)
Supplement: Supplementary file 1 — ac3c01364_si_001.pdf [file ac3c01364_si_001.pdf]

Supporting Information

The determination of phosphoethanolamine in urine with HPLC-ICPMS/MS using 1,2-hexanediol as a chromatographic eluent

Basam Lajin[a,b*], Walter Goessler[b]

[a]Institute of Chemistry, ChromICP, University of Graz, Universitaetsplatz 1, 8010 Graz, Austria

[b]Institute of Chemistry, Analytical Chemistry for the Health and Environment, University of Graz, Universitaetsplatz 1, 8010 Graz, Austria

Email address: bassam.lajin@uni-graz.at

Table of contents

Supplementary table S1. Comparing ESIMS/MS with ICPMS/MS ……………..S1

Supplementary table S1. Comparing the urinary concentrations of phosphoethanolamine between the HPLC-ICPMS/MS and HPLC-ESIMS/MS in 16 urine samples

	**ESIMS/MS (µg P L^{-1})**	**ICPMS/MS (µg P L^{-1})**	**Difference %**
U1	0.63	0.64	1.3
U2	0.53	0.50	-4.0
U3	0.47	0.46	-1.1
U4	0.85	0.86	1.7
U5	1.09	1.2	10.8
U6	0.55	0.49	-12.4
U7	2.11	1.83	-15.4
U8	0.48	0.51	4.9
U9	0.72	0.75	3.9
U10	1.65	2.0	19.3
U11	0.86	0.84	-1.9
U12	1.64	1.5	-12.3
U13	0.39	0.33	-18.3
U14	0.38	0.38	1.0
U15	1.13	1.1	-0.8
U16	1.09	0.99	-10.0
